# Ecological Risk Assessment of Geological Disasters Based on Probability-Loss Framework: A Case Study of Fujian, China

**DOI:** 10.3390/ijerph20054428

**Published:** 2023-03-01

**Authors:** Leli Zong, Ming Zhang, Zi Chen, Xiaonan Niu, Guoguang Chen, Jie Zhang, Mo Zhou, Hongying Liu

**Affiliations:** 1 Nanjing Center, China Geological Survey, Nanjing 210016, China; 2 Key Laboratory of Watershed Eco-Geological Processes, Ministry of Natural Resources, Nanjing 210016, China

**Keywords:** ecological risk assessment, geological disasters, Fujian, hazard, random forest

## Abstract

Geological disaster could pose a great threat to human development and ecosystem health. An ecological risk assessment of geological disasters is critical for ecosystem management and prevention of risks. Herein, based on the “probability-loss” theory, a framework integrating the hazard, vulnerability, and potential damage for assessing the ecological risk of geological disasters was proposed and applied to Fujian Province. In the process, a random forest (RF) model was implemented for hazard assessment by integrating multiple factors, and landscape indices were adopted to analyze vulnerability. Meanwhile, ecosystem services and spatial population data were used to characterize the potential damage. Furthermore, the factors and mechanisms that impact the hazard and influence risk were analyzed. The results demonstrate that (1) the regions exhibiting high and very high levels of geological hazard cover an area of 10.72% and 4.59%, respectively, and are predominantly concentrated in the northeast and inland regions, often distributed along river valleys. Normalized difference vegetation index (NDVI), precipitation, elevation, and slope are the most important factors for the hazard. (2) The high ecological risk of the study area shows local clustering and global dispersion. Additionally, human activities have a significant influence on ecological risk. (3) The assessment results based on the RF model have high reliability with a better performance compared with the information quantity model, especially when identifying high-level hazard areas. Our study will improve research on the ecological risk posed by geological disasters and provide effective information for ecological planning and disaster mitigation.

## 1. Introduction

Geological disasters (landslides, debris flows, etc.) often cause huge losses to life and property and seriously threaten the survival of human beings due to their suddenness and unpredictability. Geological disasters occur frequently in China and the resulting damage is particularly severe. In 2020, China experienced 7840 geological disasters resulting in direct economic losses of 5 billion yuan, according to statistical data [1]. In addition to threatening lives and property, geological disasters can damage the structure, function, safety, and health of the regional ecosystem, thereby affecting the natural foundation of the social economy, ecological civilization, and human development [2].

Ecological risk assessment (ERA) is an estimation of the likelihood that adverse ecological effects may occur when within a hazardous environmental state [3,4]. Quantifying the ecological risk could provide scientific information for government departments, formulating policies about risk prevention, ecosystem management, and sustainable development. In regional ecological risk assessments, the causes of ecological disturbances generally include human activities such as pollutant discharge and land use change, but may also include natural disasters, such as geological disasters, drought, and floods [5,6,7,8]. Among these, natural disasters, especially geological disasters, are one of the main sources of ecological risk and have become a frequent research topic for scholars [9,10].

For the ecological risk of geological disasters, the risk receptor is usually the ecosystem, though the impact of disasters on human society is also considered [11]. Currently, the ERA models related to geological disasters mainly include the relative risk model (RRM), the probability-loss model, and the landscape indices of ecological risk [12,13,14,15]. Among these, the probability-loss model is commonly adopted because of its effectiveness and efficiency [16]. This method constructs two or three dimensions by considering the probability of geological disasters, the sensitive response to geological disasters, and the potential loss of receptors [10,11,17]. The methods for describing these aspects are diverse. The probability of geological disasters is normally evaluated by data-driven quantitative models, such as the logistic model [18,19,20], information quantity model [21], and machine learning model [22]. In particular, the application of a machine learning model for geological hazards is rapidly increasing due to its ability for accurate assessments and predictions, compared with other models [23]. However, only a few scholars have assessed the hazard of geological disasters by integrating the machine learning models to construct ecological risk assessment models [17]. Additionally, in terms of damage, scholars focus on the loss of ecosystem services, which can be quantified based on multiple models or indices, and do not give enough attention to populations that are directly exposed to the disaster environments [24]. Furthermore, most studies have either focused on the loss of human life and property, or the destruction of ecosystems caused by geological disasters. Few studies have considered the two together.

Fujian, which is located in the southeast coastal area of China, is a typical mountainous province. Due to strong geological tectonic activities, complex topography, and changeable climatic conditions, the occurrence of geological disasters such as debris flows and landslides in Fujian is high. Several studies have evaluated the hazard or susceptibility of geological disasters in Fujian [21,25]. However, these studies have focused on the sensitivity of the disaster-breeding environment, and few studies concentrate on the comprehensive assessment of ecosystem risks for geological disasters. In this paper, we established a improved framework based on hazard, vulnerability, and damage, by integrating remote sensing and GIS techniques, to evaluate the ecological risk posed by geological disasters in Fujian. Hence, the objectives of this study are to (1) introduce the advanced random forest machine learning classifier on a geological disaster hazard assessment; (2) adopt landscape indices to analyze vulnerability; and (3) quantify the potential loss by integrating ecosystem services and the population. It is expected that the assessment can enrich the theories and indicator systems as they pertain to ecological risk and provide support for government departments’ disaster prevention as well as control. Moreover, the framework proposed by the study could provide a reference for other regions that face the threats imposed by geological disaster.

## 2. Materials and Methods

### 2.1. Study Area

**Figure 1:**
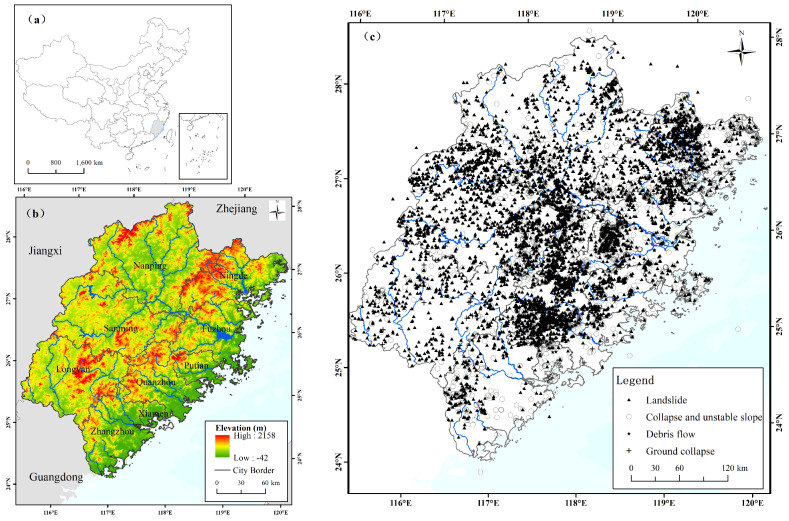
(**a**) Location of the study area in China; (**b**) a digital elevation model (DEM); (**c**) distribution of geological disasters in the study area.

With its position at 116° E–120° E and 22° N–28° N, Fujian Province is located on the southeast coast of China, having an area of approximately 126,000 square kilometers (Figure 1). There are widely distributed hills and mountains, with areas covering more than 80% of the land. The relief of the study area is low in the southeast, and high in the center and northwest. The area is the transitional area between southern and northern subtropical China. The annual average temperature is around 16–20 ℃, while the annual average rainfall is 1400–2000 mm [26]. The precipitation has a pronounced seasonality and is concentrated in summer monsoons (late June to early September). Great hydrothermal conditions have given rise to the proliferation of vegetation in the area, with a forest cover of around 65%. The river systems within the study area include the main course and tributaries of the Min River, Dai River, and Jiulong River.

The geomorphological characteristics of mountains and hills make the ecological environment of Fujian congenitally fragile. The geological composition of the area is intricate and the rock is weathered badly.The predominant soil types consist of granite weathering materials, such as red loam, brick red loam, and yellow loam, which exhibit limited water retention capability and poor corrosion resistance. Geological disasters are frequent and concentrated from May to August each year in the area. The spatial distribution of various types of geological disasters in Fujian Province is shown in Figure 1c. To date, the types of geological disasters are mainly landslides, collapses, and unstable slopes, of which the number of landslides accounts for approximately 70% of the total geological disasters, while collapses and slope instability account for approximately 27%. These geological disasters imposed a great danger on human life and property. As in Fujian, the economic activities in the study area display a marked spatial heterogeneity. The coastal areas are economically developed and densely populated compared to the inland areas.

### 2.2. Data Sources

The data used in this study include a number of datasets on land use, geological disasters, normalized difference vegetation index (NDVI), digital elevation model (DEM), precipitation, river, lithological, population, net primary productivity (NPP), evapotranspiration, rainfall erosivity, and soil erodibility data. The DEM was obtained from the dataset ASTER GDEM V2, which has a resolution of 30 m (http://srtm.csi.cgiar.org/ accessed on 15 September 2022). Slope and aspect data were extracted via DEM using ArcGIS software. The lithology comes from the digitized 1:250,000 geologic map of 2020 in Fujian Province. The geological disasters were provided by the Nanjing Center of China Geological Survey for visual interpretation. Lithological data were reclassified into five groups, as follows, based on the digitalized 1:2.5 million geological maps [27]: (1) Dolomite, thick stratified rhyolite, etc.; (2) Quartz sandstone, silastic conglomerate, etc.; (3) Pyroclastic rocks, metamorphic rocks, etc.; (4) Mudslate, coal seam, etc.; (5) Clay, loose accumulation of sediments, etc. Precipitation data were downloaded from the National Earth System Science Data Center (https://gre.geodata.cn/ accessed on 11 August 2022). This dataset was generated in China by the Delta spatial downscaling scheme based on the global 0.5° climate dataset published by the Climatic Research Unit (CRU) and the global high-resolution climate dataset published by WorldClim [28]. NDVI is a widely used remote sensing index for quantifying and monitoring the vegetation coverage, which is calculated using near-infrared and red reflectance bands. The formula for NDVI is (NIR − Red)/(NIR + Red), where “NIR” refers to the near-infrared band, and “Red” refers to the red band of the imagery. In this study, NDVI was calculated based on Sentinel-2A Level-2A images in 2020. These images were obtained via the Copernicus Open Access Hub (https://scihub.copernicus.eu/ accessed on 21 September 2022) and underwent cloud masking procedures. The 10 m land cover maps of Fujian in 2020 were downloaded from the ESA website (https://esa-worldcover.org/en/data-access accessed on 21 September 2022) [29]. We reclassified the land use types into 7 categories: forest, shrubland, grassland, cropland, water, build-up, and bareland. The spatial distribution of the population in 2020 was acquired from WorldPop (https://hub.worldpop.org/ accessed on 15 September 2022) with a resolution of 3 arc (approximately 100 m). NPP and evapotranspiration data were acquired from MOD17A3HGF V6 and MOD13 products, respectively. The spatial resolution for both datasets is 500 m. The rainfall erosivity and soil erodibility data were obtained from the National Earth System Science Data Center, National Science & Technology Infrastructure of China (http://www.geodata.cn accessed on 17 September 2022) [30]. All spatial data were resampled with 30 m resolution using the cubic convolution method, and the projection was unified as Transverse Mercator.

### 2.3. Methods

#### 2.3.1. Research Framework

The ecological risk of geological disaster can be defined as the product of the probability of the disaster and loss caused to the ecosystem and humans once the disaster occurs [31]. Herein, the two-dimensional model was extended and a framework that integrated hazard, vulnerability, and potential damage assessments was proposed. This study introduced the advanced machine learning model on hazard assessment. Additionally, ecosystem loss and population loss were both included in the potential loss assessment, considering the great damage of geological disasters to human property and life.

**Figure 2:**
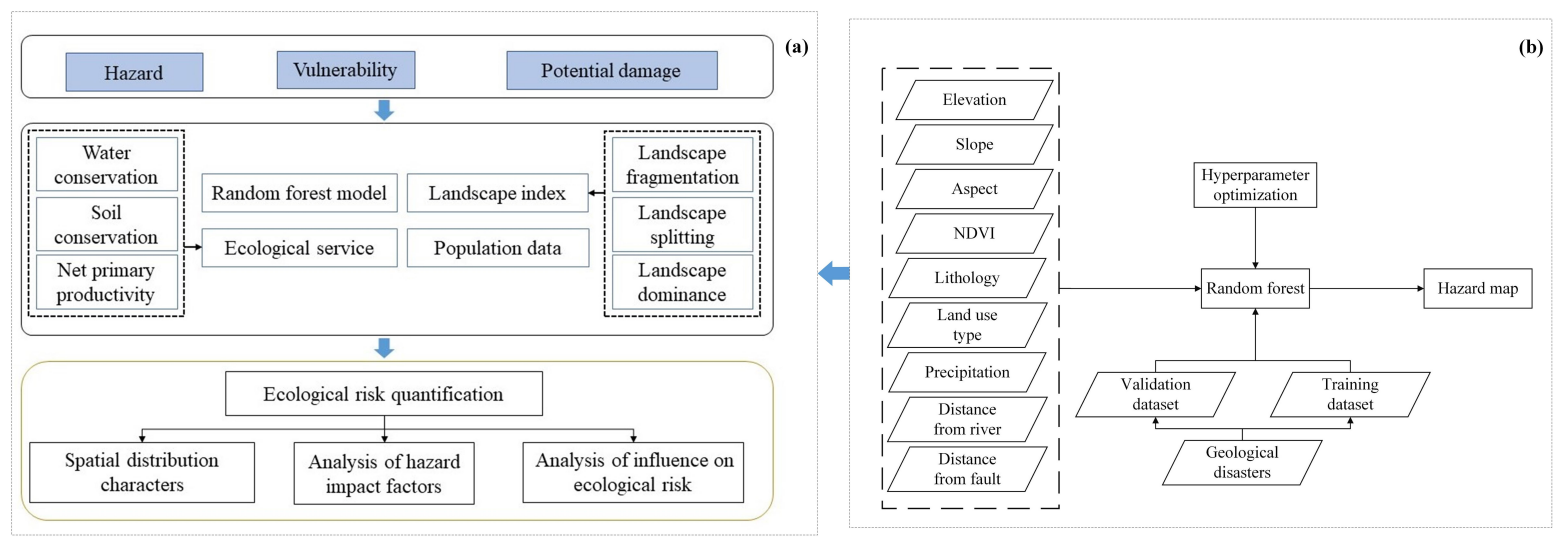
The flowchart of (**a**) ecological risk assessment framework and (**b**) hazard assessment of geological disasters.

The ecological risk assessment framework for geological disasters proposed in this paper is shown in Figure 2b. The ecological risk was quantified based on hazard, vulnerability, and damage assessment. Among them, the hazard of geological disasters was evaluated based on a random forest model combined with multiple index factors, and the vulnerability was calculated by landscape indices. The potential damage, starting from multiple risk receptors, was represented by both ecosystem services and population. The quantitative assessment results of the ecological risk were calculated by the equation “$Risk=Hazard\times Vulnerability\times Damage^{}$”. Based on this, the spatial distribution characteristics of hazard, vulnerability, and potential damage were analyzed.

#### 2.3.2. Quantifying the Hazard Based on Random Forest Model

The overall method flowchart accompanying the hazard evaluation of geological disasters is given in Figure 2b. First, geological disaster points and the risk assessment index system were prepared, and their spatial database was generated. Second, the values of each conditioning factor were extracted by overlapping the training dataset of the geohazard, as the input features of the RF model. Third, the hazard map was obtained using a well trained RF model. Moreover, the accuracy of the model was evaluated by using the receiver operating characteristic (ROC) curve and frequency ratio analysis.

In this paper, 5970 historical geological disasters within the study area were selected as positive samples. In the meanwhile, the non-geological disaster areas were generated by removing the 500 m radius buffer areas around all the geohazard points as well as the river land [32]. Moreover, 5970 non-geological disaster points were randomly selected inside this area as the negative samples. Finally, the positive and negative samples were combined to form the training dataset.

**Figure 3:**
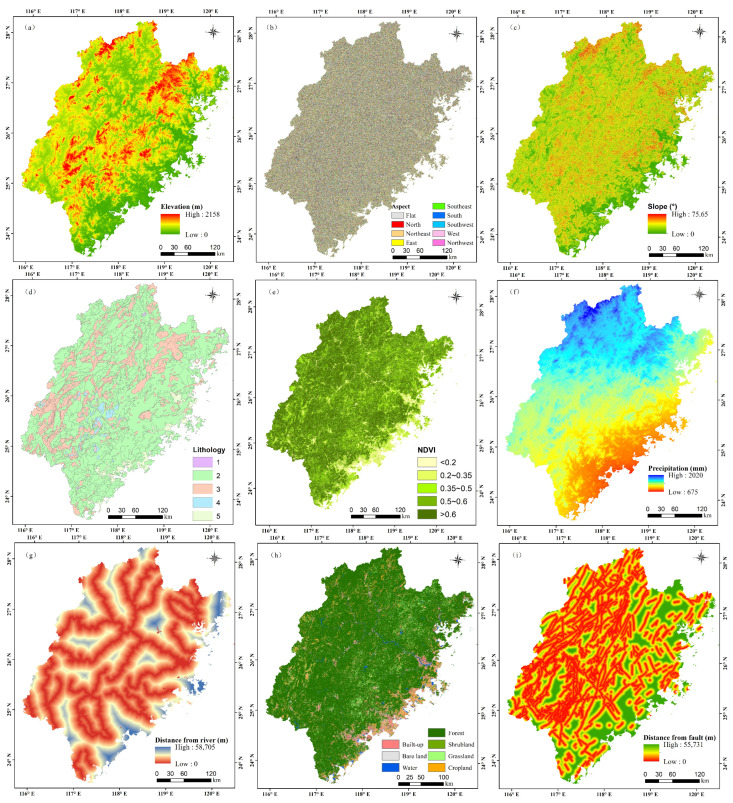
Conditioning factors of hazard evaluation.

The selection of suitable conditioning factors is essential for hazard assessment. Nine factors were chosen for hazard assessment in this study according to the site survey and former studies. These factors include elevation, slope, precipitation, NDVI, aspect, land use type, lithology, distance from the fault, and distance from the river [11,33]. The spatial distribution of the hazard assessment indicators is shown in Figure 3.

#### Random Forest Model

The random forest (RF) algorithm is a machine learning technique that was first introduced by Leo Breiman and Adele Cutler [34]. The core concept of this method involves the construction of multiple classification and regression trees (CART) that are independently grown. The method begins by obtaining different training sets through random sampling via the bootstrap method. In the growth process of each tree, the optimal feature selection and node division are performed based on the Gini coefficient, a widely used measure of node impurity in the CART analysis. The final classification is determined through a voting process, with the category receiving the most votes is selected as the result. As an ensemble learning algorithm, the random forest (RF) has been widely recognized for its exceptional generalization ability, high prediction accuracy, and robust stability [35].

The hyperparameter optimization plays a critical role in the performance of machine learning models, which is particularly true for the random forest (RF) algorithm. As reported in previous studies, the main parameters that have been demonstrated to affect the performance of RF are the number of decision trees and the size of the features selected for each tree [36]. To optimize these hyperparameters, a grid search method based on the out-of-bag (OOB) error value was employed in this study. The implementation of the RF model was conducted using the scikit-learn library, a widely used python-based data analysis toolkit. In order to evaluate the importance of each feature, the mean impurity decrease derived by the RF model was used. The optimization of the parameters was performed by a grid search, and the final accuracy was verified through five-fold cross-validation (CV). In this study, the number of trees was set to 100 and the number of features was set to 5.

#### Model Validation and Accuracy Analysis

The accuracy assessment and validation of the models are important for hazard analysis. In this study, two different techniques, the ROC curve and frequency ratio analysis [37], were used to measure the accuracy of the model. Among them, the ROC curve is widely used to test the accuracy of the geological hazard evaluation, which represents the relationship between simulated data and actual data. The area under the curve (AUC) could reflect the precision of the model directly, with values ranging from 0.5 to 1. The larger the value is, the closer the fitted value is to the actual value, and the higher the prediction accuracy of the model [38]. In the case of the frequency ratio method, the hazard map generated by the model was divided into five levels based on the natural break point method, corresponding to risk levels from very low to very high. Next, a relative frequency ratio analysis was performed on the hazard map by overlaying the geological disaster points.

Moreover, to further evaluate the effectiveness and reliability of the RF model, as applied to the geological hazard assessment, it is necessary to compare the RF model with other geological hazard assessment methods. The study compared the performance of the RF and the information quantity model to assess the hazard of geological disasters. The prediction result was obtained by comparing geohazard grid cells in the validation dataset (2551 cells that were not used in the training dataset) with the two hazard maps.

#### 2.3.3. Vulnerability Assessment via Landscape Pattern Indices

Ecosystem vulnerability could reflect how likely an ecosystem is to change in response to external disturbances, structure, composition, and other ecological characteristics. Landscapes change slowly within an ecosystem but can change rapidly when disturbed by external factors. Landscape patterns reflect the manner and extent of human influence on natural ecosystems. These ecological impacts are regional and cumulative in nature and can be reflected by the structure and composition of ecosystems. The landscape pattern index (LPI) quantifies the interactions between landscape heterogeneity and ecological processes and is suitable for vulnerability mapping at larger spatial scales [39].

In this study, the patch density (PD), landscape division index (DIVISION), and landscape disturbance index (LDI) were selected with reference to the basic principles and technical approaches of landscape index selection [40,41]. *PD*, *LDI*, and *DIVISION* were used to construct a composite index to measure the vulnerability of regional ecosystems under the geohazard disturbance. The three landscape indices were calculated and standardized, and their mean values were obtained. The formulas related to the vulnerability indices are shown below.

(1) $$VI=\frac{\left(LDI+PD+DIVISION\right)}{3}$$

where VI is the composite vulnerability index; the higher the value of this index is, the less stable the regional ecosystem and its resistance to external disturbance. PD stands for the fragmentation of the landscape ecosystem, the higher the fragmentation, the less resilient the ecosystem is to disturbance. LDI can reflect the degree of disturbance to different landscape ecosystems. The LDI can be calculated from the landscape fragmentation index $\left(C_{i}\right)$, the $\left(S_{i}\right)$, and the landscape dominance index (D).

(2) $$LDI=aC_{i}+bS_{i}+cD$$

(3) $$C_{i}=\frac{N_{i}}{A_{i}}$$

(4) $$S_{i}=\frac{A}{2A_{i}}\sqrt{\frac{n_{i}}{A}}$$

(5) $$D=ln\left(m\right)+\sum_{i=1}^{n}\left(\frac{A_{i}}{A}\right)\times ln\left(\frac{A_{i}}{A}\right)$$

where *a*, *b*, and *c* are assigned as 0.5, 0.3, and 0.2,respectively, according to previous studies. $N_{i}$ is the number of patches of the *i*th landscape. $A_{i}$ is the area of the *i*th landscape (km${}^{2}$); *A* is the total area of all landscapes; m is the number of the landscape types.

When calculating ecological risk in the landscape, the size of the landscape analysis unit should be 2 to 5 times the average patch size [42]. We divided the study area into 0.5 km × 0.5 km grids, with a total of 546,268 grid cells, as vulnerability assessment units.

#### 2.3.4. Calculation of Potential Damage

The ultimate receptors of ecological risk are not only humans themselves but also the component structures of ecosystems. The study used ecosystem and the exposed population as ecological risk receptors. As the benefits that humans derive from ecosystems, ecosystem services mainly include provision, regulate and support services such as soil conservation, water conservation, and biodiversity conservation. A specific ecosystem pattern could maintain the ecological services. Once this pattern is disrupted, the ecosystem services could decline, and finally increase the ecological risk.

Considering the ecological environment status of the study area, three ecosystem services, including water conservation, soil conservation, and the net primary productivity (NPP) of vegetation, were selected to represent the potential ecological losses. In this study, the three layers were standardized by range and then spatially superimposed as the potential loss of the ecosystem after being stressed by geological disasters. In addition, population distribution data were used to represent the population exposure after the occurrence of geological disasters. After the range standardization of the potential ecosystem loss and population exposure, we obtained the final potential loss through the spatial overlay tool in ArcGIS software.

#### Water Conservation

The calculation of water conservation was based on the water balance method [43].

(6) $$Q_{wr}=\sum_{i=1}^{n}\left(P_{i}-R_{i}-ET_{i}\right)\times 10^{-3}$$

where $Q_{wr}$ represents the amount of water conservation (m${}^{3}$/a); $P_{i}$ represents precipitation (mm/a); $R_{i}$ stands for surface runoff (mm/a); $ET_{i}$ represents evapotranspiration (mm/a); and *i* represents the type of ecosystem service.

#### Soil Conservation

The calculation of soil conservation is based on the Revised Universal Soil Loss Equation (RUSLE), which calculates the difference between potential soil erosion and actual soil erosion, thereby determining the amount of soil conservation achieved [44,45].

(7) $$SE_{p}=R\times K\times LS$$

(8) $$SE_{r}=R\times K\times LS\times C\times P$$

(9) $$SOR=SE_{p}-SE_{r}$$

where SOR is the amount of soil conservation (t·hm${}^{-2}\cdot$a${}^{-1}$), $SE_{p}$ represents the potential erosion, $SE_{r}$ represents the actual erosion, *R* represents the rainfall erosivity factor (MJ·mm·hm${}^{-2}\cdot$h${}^{-1}\cdot$a${}^{-1}$), *K* is the soil erodibility factor (t·hm${}^{2}\cdot$h·hm${}^{-2}\cdot$MJ${}^{-1}\cdot$mm${}^{-1}$), LS is the topographic factor and can be calculated from a DEM [46] (Appendix A), *C* is the vegetation factor [44,47] (Appendix A), *P* is the management factor, and the value was assigned based on different land use types by referring to previous studies [48,49,50].

## 3. Results

### 3.1. Analysis on Hazard of Geological Disasters

#### 3.1.1. Validation of Results for Hazard Assessment

**Figure 4:**
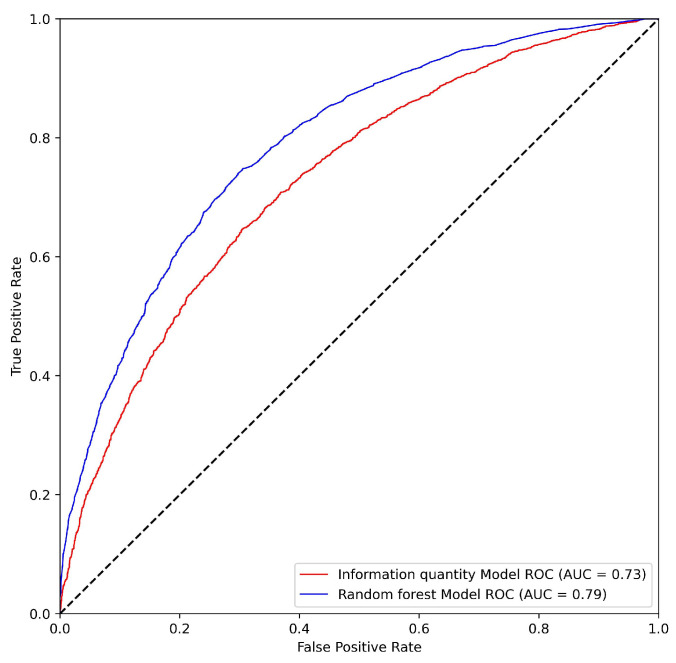
Comparison of ROC values between the random forest model and information quantity model.

The area under the ROC curve (AUC) value of the hazard result based on the RF and information quantity model was 0.79 and 0.73, respectively (Figure 4). The results indicated that the prediction result of RF model had high credibility and could be used for the evaluation result of a geological disaster. The AUC value of the RF model is higher than that of the information model.

**Figure 5:**
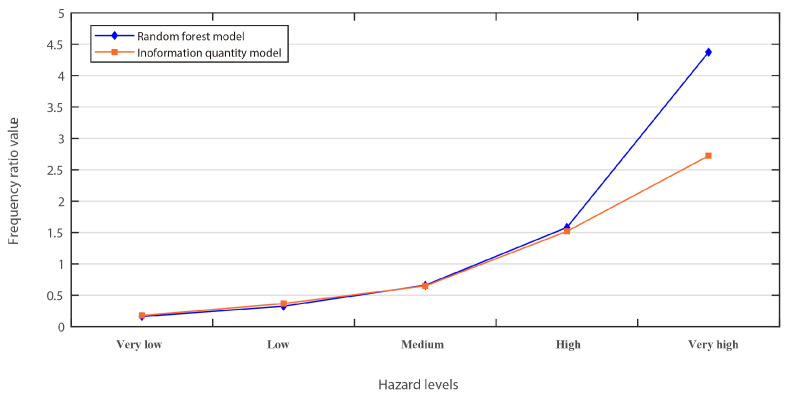
Frequency ratio plot of four hazard levels of RF and information quantity models.

The second approach was employed to assess the accuracy of the model, and the efficiency of its predictive power was the frequency ratio method. This method is based on the theoretical premise that the frequency of a phenomenon will gradually increase as hazard levels progress from very low to very high. The results of this analysis are presented in Figure 5, which demonstrates that the frequency ratio values of both the random forest (RF) model and the information quantity model increase as hazard levels progress from very low to very high. Furthermore, the characteristics of the five hazard levels for the two results obtained from the RF and information quantity models are shown in Table 1. The frequency values of existing geological disasters that fell into the very high level are 9.12 and 5.78 for the RF and information quantity models, respectively. There are few differences in other levels of the frequency value between the two models. A further analysis was carried out to explain the better performance of the RF, especially in the hazard assessment of high-level areas. For the RF model, 72% of the historical verification geological disaster points fall in high and very high hazard regions, while for the information quantity model, it is 69%. Thus, compared with the information quantity model, the RF model has better fitting results and is more suitable for a hazard assessment in the study area.

#### 3.1.2. Spatial Distribution of Hazard

**Figure 6:**
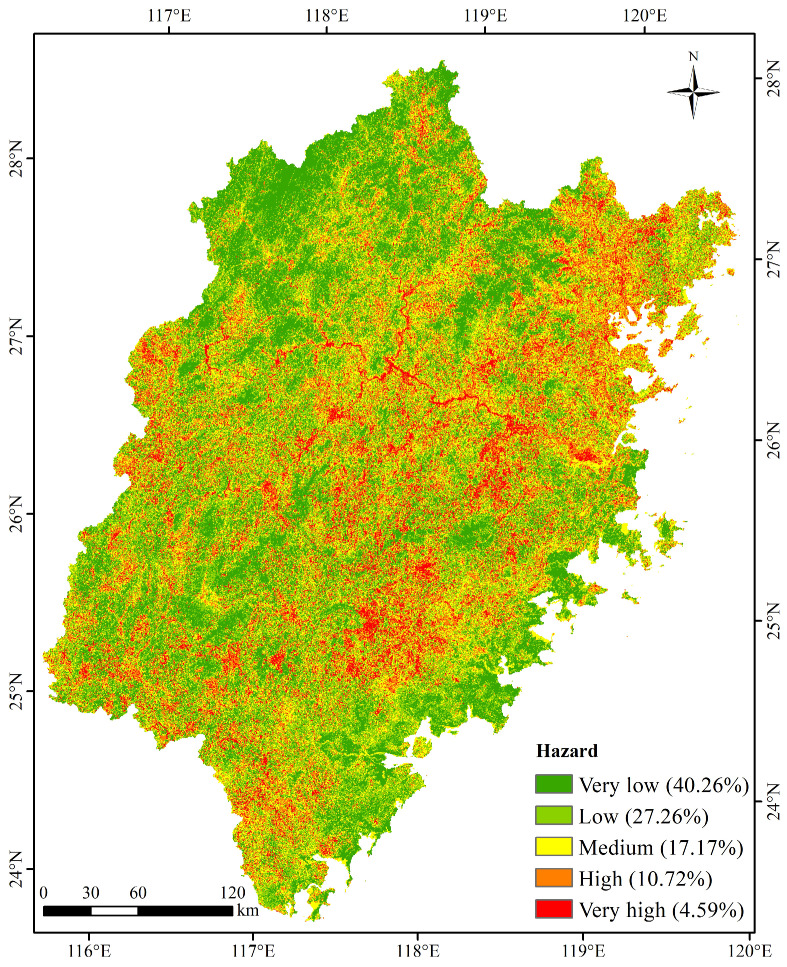
Spatial distribution of hazard of geological disasters.

In this study, 70% (5970 geological disaster points) of the 8528 geological disaster points were randomly selected for the training of the RF model, and the remaining points were used for the verification of the hazard assessment results. Based on the RF model, the hazard assessment results for geological disasters were obtained. The AUC value of the hazard result was 0.73, indicating that the prediction result had a high credibility and could be used as to evaluate geological disasters. The result was divided into five levels using the breakpoint method. Figure 6 shows the spatial distribution characteristics of hazard of geological disasters in Fujian Province. The high and very high levels of geological hazards in Fujian Province account for 10.72% and 4.59% of the total land area, respectively. In terms of spatial patterns, the areas with high and very high levels are mainly distributed in the northeastern and central parts of the study area. The distribution pattern of the linear cluster along the river valley is presented in the northeastern part of the study area. The hazard levels of the southeast coastal area and northwest inland area are low.

### 3.2. Spatial Distribution of Vulnerability and Potential Damage

#### 3.2.1. Spatial Distribution Characteristics of Vulnerability

**Figure 7:**
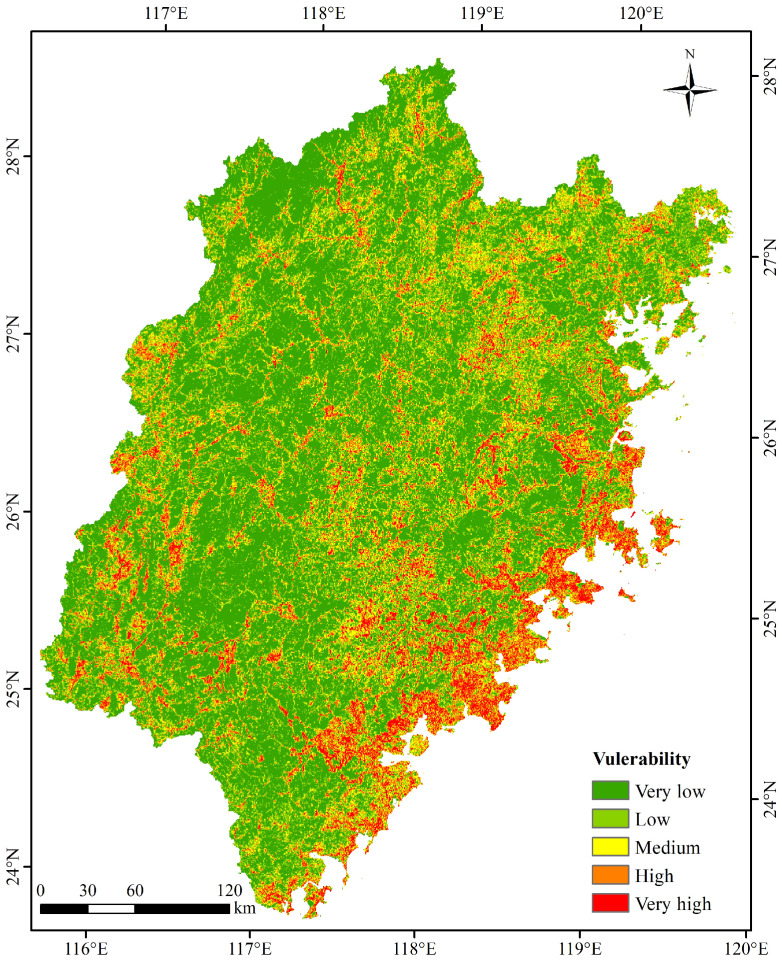
Spatial distribution characteristics of vulnerability.

According to the vulnerability assessment model presented in Section 2.3.3, the vulnerability value of each unit was calculated. Based on the natural breakpoint method, the vulnerability score was divided into five levels: very low, low, medium, high, and very high. The spatial distribution of vulnerability is illustrated in Figure 7. Regions of high vulnerability are primarily concentrated in the economically developed southeast coastal region of the study area. These areas are characterized by high land use intensity, landscape fragmentation, and a threatened ecosystem stability, resulting in a highly fragile ecosystem.

#### 3.2.2. Spatial Distribution Characteristics of Potential Damage

**Figure 8:**
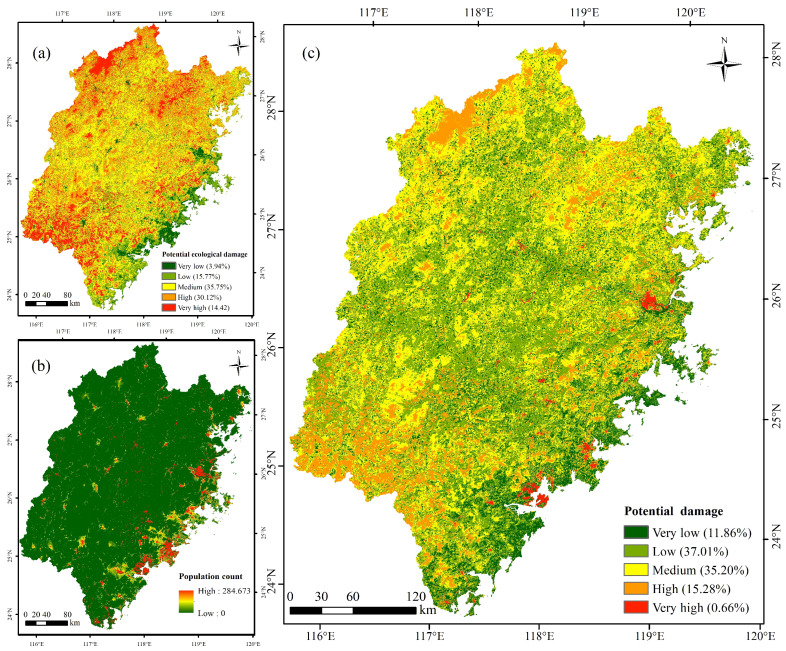
Spatial distribution of (**a**) potential ecological damage; (**b**) population count; (**c**) potential damage of geological disasters in the study area.

In this study, the potential damage was calculated by overlaying the ecological damage layer and the population exposure layer. All the layers were categorized into five levels (very low, low, medium, high, and very high) with the natural break point method. The spatial distribution characteristics are shown in Figure 8. The potential ecological damage in the study area was mainly high and medium. The area with the highest value of ecological loss is mainly distributed in Wuyishan city in the northwest of the study area and Longyan city in the southwest. These areas have good hydrothermal conditions and lush vegetation, resulting in strong net primary production. Meanwhile, the forest cover is high, and the amount of water conservation is large; therefore, it has strong ecosystem services. However, the southeast coastal area has strong human activities, low vegetation coverage, and weak ecosystem service ability, so the value of ecological loss is low. The distribution of the population exposure is the opposite. Areas with high and very high exposure were concentrated in the southeast coastal areas.

In terms of total potential losses, the regions with higher grades are mainly located in the surrounding areas of Fuzhou and Xiamen. These areas have low vegetation cover and weak ecosystem services. However, due to their strong human activities and high population density, the potential loss of people and property under the stress of geological disasters is great. In addition, due to the high ecological loss in the northwest and southwest of the study area, the overall loss is also at a high level.

### 3.3. Ecological Risk of Geological Disasters

#### 3.3.1. Spatial Pattern of Ecological Risk

**Figure 9:**
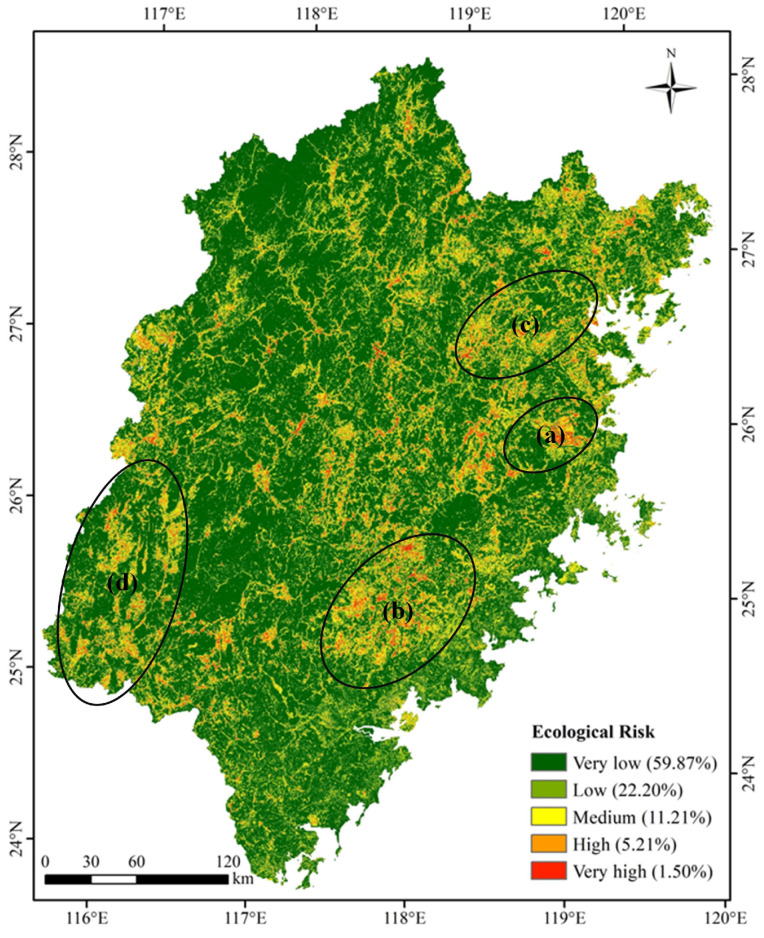
Spatial pattern of ecological risk. The circles are the typical regions with high ecological risk.

Based on the hazard, vulnerability, and potential loss of geological hazards, the ecological risk of geological disasters in Fujian at the grid scale was obtained by an equal weight multiplication. The ecological risk of geological disasters was divided into very high, high, medium, low, and very low levels according to the natural breakpoint method (Figure 9).

The proportion of areas with high and very high ecological risk levels in Fujian is relatively low, accounting for 5.21% and 1.5% of the total land area, respectively. Overall, it shows a distribution pattern of local aggregation and global dispersion. Specifically, the west, southwest, east, and southeast of the study area were all distributed with high risk levels. The clustered high and very high risk areas are mainly located around the West River, Min River, and Ting River valleys. These are areas of intense human activity and fragile ecology. Meanwhile, the potential damage due to geological disasters is relatively large in densely populated areas. Medium risk level areas are mainly located in the periphery of the high risk zone and along the river.

#### 3.3.2. Mechanisms of Influence on Ecological Risk

The analysis on the genesis of the ecological risk posed by geological disasters is crucial for risk prevention and control. In this paper, four areas with high ecological risk in Fujian were chosen as typical cases to analyze the mechanisms of influence on ecological risk, combining with the results of ecological risk assessment and physical conditions. The high-risk areas (Figure 9) were as follows: (a) the area near Fuzhou Plain in the eastern part of the study area; (b) the southeastern part of Daiyun Mountain; (c) the southwest edge of Jiufeng Mountain in the northeast part of the study area; (d) the valley area of the Ting River watershed.

The area near the Fuzhou Plain (a) has high hazard and high vulnerability. This area is in a river valley with low vegetation coverage, and its rock strata are mainly soft rocks containing viscous and silty grains, which, objectively, is a disaster-prone environment. What is more, dense anthropogenic activities make the ecosystem extremely vulnerable with fragmented patches. Moreover the potential damage in this area is large due to the dense population. Hence, the overall ecological risk level is high.

The southeastern part of Daiyun Mountain (b) mainly includes Anxi and Yongchun Counties, which have high levels of hazard and vulnerability. This mountainous area has a high elevation with a steep slope. Moreover, the average annual precipitation in this area is approximately 1600 mm, which is conducive to the development of geological disasters. In addition, Anxi and Youngchun Counties have numerous tea gardens, as important regions for tea production. According to the third land survey results, the garden area of Anxi County accounted for 27% of the county’s land area. Most tea plantations are located on the slopes of hilly and mountainous areas. Frequent cultivation and planting activities will aggravate soil and water loss in the area and cause instability of the rock and soil mass. When encountering geological disasters, the anti-interference ability of this area is poor, and the ecological environment is fragile. Therefore, the ecological risk level in this region is high.

The southwest edge of Jiufeng Mountain (c) is a typical hilly area. This region has a high hazard of geological disasters with rugged and complex terrain and high rainfall intensity. Meanwhile, there are patches of orchards and scattered paddy fields in the area, which makes the ecological environment vulnerable, to a certain extent. The valley area of the Ting River watershed (d) is frequently impacted by human activities, including engineering and farming, which leads to the high vulnerability in the region. Moreover, potential losses are high in the valleys because of the high ecosystem services and concentrated population, with good hydrothermal conditions.

Generally, the causes and mechanisms of the high ecological risk in the study area have significant spatial variability. Apart from the objective environment that breeds disasters, human activities, including farming and engineering, also play important roles in the high ecological risk.

## 4. Discussion

### 4.1. Analysis of the Hazard Impact Factors

**Figure 10:**
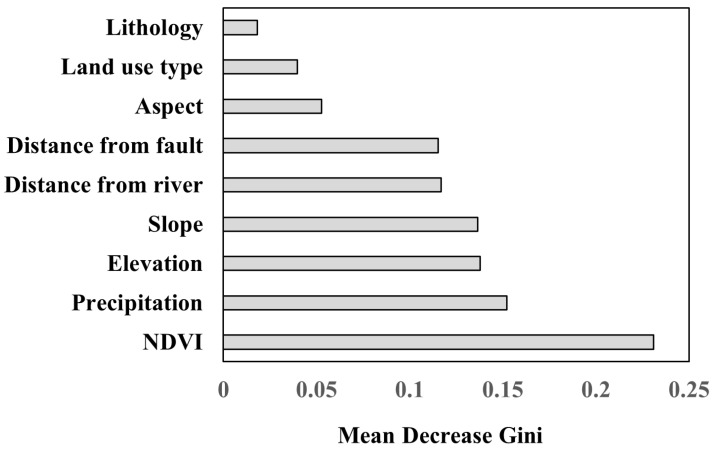
Mean decrease Gini of variables assigned by the random forest.

It is imperative to investigate the significance and mechanism of conditioning factors in understanding their impact on the geological hazard, as it provides valuable information for the prediction and prevention of geological disasters. In this study, the Mean Decrease Gini of the RF model was utilized to evaluate the importance of the nine conditioning factors [51]. As depicted in Figure 10, NDVI was found to be the most influential factor, followed by precipitation, elevation, slope, distance from the river, distance from the fault, aspect, land use type, and lithology.

**Figure 11:**
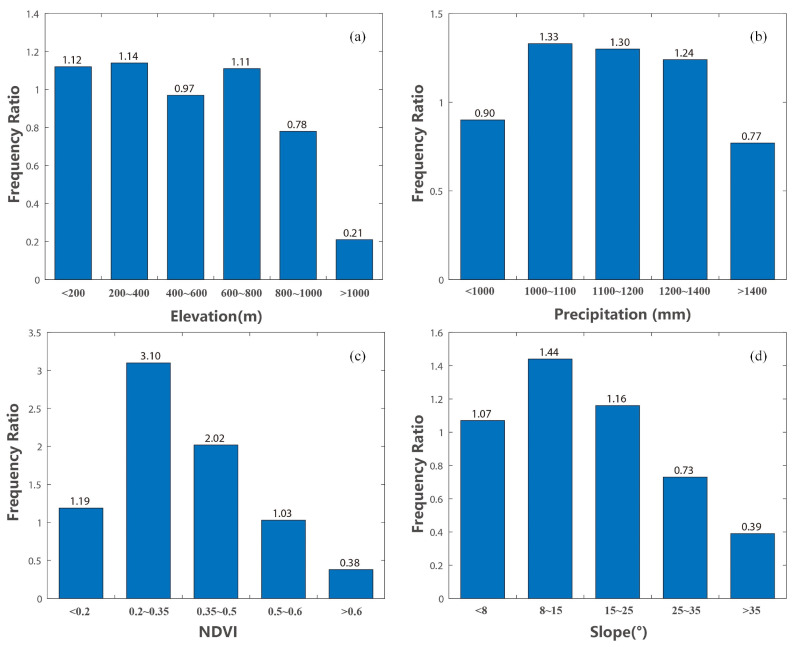
Frequency ratios of typical conditioning factors: (**a**) NDVI; (**b**) precipitation; (**c**) evaluation; (**d**) slope at different intervals.

Furthermore, the top four factors, namely, NDVI, precipitation, elevation, and slope, were selected to analyze the impacts of the factors on the hazards. The layers of these four factors were reclassified into different intervals, and then the geological disaster points were overlaid to calculate the corresponding frequency ratio for each interval. Figure 10 shows the frequency ratios of these typical conditioning factors at different intervals, which reflects the partial effects on geological disaster hazards. It can be observed from the figure that the frequency ratio value first increased and then decreased as the NDVI continued to rise. The areas with NDVI values less than 0.2 are mostly built-up areas with relatively few geological disaster points. In areas with NDVI values exceeding 0.2, the frequency of geological disasters increases proportionally with the decreasing vegetation coverage. Rainfall is the second most important conditioning factor. As shown in Figure 11b, the frequency of geological disasters is higher when the precipitation is between 1000 mm and 1400 mm. This range of precipitation can cause soil erosion, which can increase the likelihood of landslides and other geological hazards. When the precipitation is higher than 1400 mm, the frequency is lower. The cause for this phenomenon may stem from the fact that regions with high precipitation levels typically exhibit a higher degree of vegetation development, which in turn serves to stabilize the soil and mitigate the occurrence of geological disasters. As the third important conditioning factor, in general, elevation is closely related to the impact of geological disasters on human activities. Geological hazards are more likely to occur in low mountainous areas with altitudes of less than 1000 m (see Figure 11c). In areas with low elevations, human activities such as house and road construction lead to unstable slopes, which are accompanied by loose soil, and tend to aggravate the occurrence of geological hazards. For the slope factor, the frequency ratio value first increased and then decreased as the slope continued. Geological disasters occur more frequently in regions with small slopes than in areas with large slopes. On the one hand, areas with low slope values tend to have strong human activities. On the other hand, the soils located in regions characterized by steep slopes exhibit a higher susceptibility to the gravity-induced displacement, which makes the formation of weak weathering layers more challenging. As a result, the occurrence of geological hazards, such as landslides, is less frequent in these areas.

### 4.2. Development Strategy for Hazard Prevention and the Improvement of the ERA

From the analysis above, the factors that influence disasters in different high-risk areas vary. Thus, the proposed risk control measures should be more targeted and adapted to local conditions. In future policy making, economic development and ecological protection should be considered simultaneously. In areas characterized by high levels of hazard, vulnerability, and potential damage, the frequency of geological disaster monitoring should be improved, and prohibited development zones should be established in some areas. For areas of high vulnerability, corresponding development protection measures, such as the reinforcement of unstable slopes, should be established. In addition, for areas with high potential ecological losses, conservation should be the primary strategy, and land development and use should be avoided as much as possible.

The current study has certain limitations that should be acknowledged and addressed in future research endeavors. Specifically, the assessment of potential damage was limited to three types of ecological services due to a lack of detailed spatial data. Furthermore, the risk assessment methodology employed in this study could be improved by incorporating uncertainty and sensitivity analyses to better understand the spatial variability of the results. In future research, it would be beneficial to consider a wider range of ecosystem services to improve the calculation of potential losses, and to conduct a more comprehensive uncertainty and sensitivity analysis to better understand the effectiveness of the assessment results.

## 5. Conclusions

In this study, the “hazard-vulnerability-potential damage” framework was used to evaluate the ecological risk of geological disasters in Fujian, and the influencing factors were further analyzed. The framework could have a broader applicability in other regions that are under the threat of geological hazards. Moreover, our results could provide a guidance and reference for the prevention and control of ecological risks. The primary conclusions are as follows:

(1) The hazard of geological disasters are mainly medium risks. The area with high and very high levels of geological hazard account for 10.72% and 4.59% of the study area, respectively. These areas are mainly distributed in the northeast and inland regions and present a striped distribution pattern along the river valley. The results of the conditioning factor importance evaluation and impact analysis of typical conditioning factors showed that NDVI, precipitation, elevation, and slope are the most important factors that may encourage the geological disaster hazard.
(2) The high ecological risk of the study area shows trends of local clustering and global dispersion. The areas with high ecological risk are mainly concentrated along the Fuzhou Plain, southeastern part of Daiyun Mountain, southwest edge of Jiufeng Mountain, and valley area of the Ting River watershed. The causes and mechanisms of the high ecological risk in the study area have significant great spatial variability, and human activities have a significant influence on the ecological risk.
(3) The geological disaster hazard assessment result based on the random forest model has a high reliability. The AUC value of the ROC curve is 0.79, and 72% of the historical verification geological disaster points fall within high hazard regions. Compared with the information quantity model, the RF model performed better in terms of the hazard assessment of geological disasters, especially regarding the identification of high-level hazard areas.

## Figures and Tables

**Table 1:** Distribution of hazard levels overlaying geological disasters of RF and information quantity models.

	Random Forest Model			Information Quantity Model		
Hazard Level	Area (km${}^{2}$)	Number of Geological Disasters /Proportion	Frequency (/km${}^{2}$)	Area (km${}^{2}$)	Number of Geological Disasters /Proportion	Frequency (/km${}^{2}$)
Very low	28,621.38	96/3.76%	0.34	10,330.91	39/1.53%	0.38
Low	31,239.9	212/8.31%	0.68	32,976.65	257/10.07%	0.78
Medium	28,747.29	395/15.48%	1.37	35,017.88	482/18.90%	1.38
High	21,083.87	695/27.24%	3.30	25,278.4	814/31.91%	3.22
Very high	12,639.22	1153/45.20%	9.12	16,602.56	959/37.60%	5.78

## Data Availability

The data that support the findings of this study are available from the corresponding author, upon reasonable request.

## Appendix A

The calculation of the topographic factor (LS) and vegetation cover factor (C) in the Revised Universal Soil Loss Equation (RUSLE) model is presented below. For the topographic factor, the equation is shown as follows:

(A1) $$LS={\left(\frac{\lambda }{72.6}\right)}^{m}\times \left(65.41sin2\beta +4.56sin2\beta +0.065\right)\;$$

(A2) $$m=\left\{\begin{array}{c} 0.2\;\;\;\;\beta <0.57\\ 0.3\;\;\;\;0.57\leq \beta <1.72\\ 0.4\;\;\;\;1.72\leq \beta <2.86\;\\ 0.5\;\;\;\;\beta \geq 2.86 \end{array}\right.$$

where LS is the slope factor of the slope length, λ is the slope length (m), and β is the slope gradient (arc degree) extracted from the digital elevation model (DEM) data.

The Vegetation cover factor (C) was calculated by different methods based on different land use types. For water, construction land and bare land, values of 0, 0.1,0.7 were assigned [47]. Moreover, for forest land, shrub land and grass land, the value was calculated as shown in Equation (A3) [44].

(A3) $$C=\left\{\begin{array}{c} 1\;\;\;fc=0\\ 0.6508\;\;\;\;0<fc\leq 78.3\%\\ 0\;\;\;fc>78.3\% \end{array}\right.$$

where fc is the vegetation cover factor.
